# How soon do single mothers have another child? A competing risk analysis of second premarital childbearing in sub-Saharan African countries

**DOI:** 10.1186/s12884-020-2850-1

**Published:** 2020-03-29

**Authors:** Clifford O. Odimegwu, Emmanuel O. Olamijuwon, Vesper H. Chisumpa, Joshua O. Akinyemi, Mwiza G. Singini, Oluwaseyi D. Somefun

**Affiliations:** 1 Demography and Population Studies Programme, Schools of Public Health and Social Sciences, University of the Witwatersrand, Johannesburg, South Africa; 2 Department of Statistics and Demography, Faculty of Social Sciences, University of Swaziland, Kwaluseni, Swaziland; 3 Department of Population Studies, School of Humanities and Social Sciences, University of Zambia, Lusaka, Zambia; 4 Department of Epidemiology and Medical Statistics, Faculty of Public Health, College of Medicine, University of Ibadan, Ibadan, Nigeria; 5 Lighthouse Trust, Kamuzu Central Hospital, Lilongwe, Malawi

**Keywords:** Non-marital childbearing, Premarital childbearing, Single motherhood, Fertility behavior, Sub-Sahara Africa, Demographic health survey

## Abstract

**Background:**

A considerable number of previous studies have examined the trends, correlates, and consequences of premarital childbearing among adolescents and young women in Africa. However, very little is known about whether and how soon single mothers have another premarital birth in sub-Saharan African countries. This study examines the timing of a second premarital birth among single mothers and assesses how it may differ across key socio-demographic variables.

**Methods:**

We pooled recent Demographic and Health Surveys from 25 sub-Saharan African countries to create a database of 57, 219 single mothers aged 15–49 years. Cumulative incidence graphs and Fine and Gray’s competing risk models were used to delineate the timing of a second premarital birth and its socio-demographic correlates.

**Results:**

More than one-third of single mothers in 16 countries have had a second premarital birth in their reproductive life. We also observed that more than 15% of the single mothers in Angola, Benin, the Republic of Chad, Liberia, Namibia, Nigeria, Sierra Leone, and Uganda, have had another premarital birth three years after the first. The incidence of a second premarital birth was significantly lower among women with secondary or higher education, compared to women with less than secondary education *(p < 0.05)* in most countries. Residence in an urban area compared to rural, was also significantly associated with a low incidence of second premarital birth in 10 countries *(p < 0.05).*

**Conclusions:**

Findings indicate a rapid progression to having a second premarital birth in some sub-Sahara African countries, particularly among socio-economically disadvantaged women. The findings suggest the need for tailored interventions for improving the quality of life of single mothers, to reduce the associated burden and consequences of having a premarital birth.

## Background

Premarital childbearing in sub-Sahara African (SSA) countries is becoming an important phenomenon—demographically and socially [1–7], in part because of its implications for single mothers, their children, and society. Garenne [8] notes that in several African countries, the median age at first marriage has risen by an average of one to two years. The age of first marriage has increased even more rapidly in several parts of Southern Africa, to more than 25 years for women [8]. Although there are speculations that the rising age of first marriage and early sexual debut coincides with higher levels of premarital childbearing [3, 9, 10], a recent study suggests that countries can achieve relatively stable, or decreasing levels of premarital childbearing, even with the increasing age at first marriage [1]. In contrast, the levels of premarital childbearing continue to rise in many sub-Saharan African countries [1].

Having a premarital birth has diverse implications, especially for unmarried mothers, rather than their male counterparts [6, 11]. For instance, in some communities, the socio-economic development of such women with regards to education and accessibility to job opportunities may be restrained [12–15]. They also tend to have higher poverty rates, fewer economic resources than married mothers, and less support from families [16–18]. Recent evidence also suggests that having a premarital birth may affect women’s marriageability in African countries [5, 15]. These pieces of evidence suggest that having a premarital birth may reduce women’s “attractiveness” and limit their access to potential partners with relatively high economic status, who may not be willing to assume the financial and parental responsibilities associated with marrying a single mother [5, 15, 19, 20]. These risks could even be worse for women with multiple premarital births.

Over the past decade, evidence on the benefits of marriage for the well-being of children in sub-Saharan Africa, its timing and events within it, has continued to mount. Previous studies have shown that the children of single mothers have higher mortality risks, and poorer health outcomes compared to children born to married mothers [7, 21, 22]. These findings may partly be because women who have a child before marriage, spend a considerable number of years as a single parent and struggle to care for their child and themselves [5]. Besides, single mothers are unlikely to reside in the same household with their children, thus limiting the amount of maternal care their children receive [21]. These and other implications of being born premaritally or having a premarital birth, necessitate the need for continued research to understand better the socio-cultural contexts in which families formed outside of marriage, continue to grow in number and perhaps size in sub-Saharan African countries.

Despite the economic hardship and the implication of premarital childbearing for children and women’s health, it remains unclear whether women would have a second premarital birth as well as the timing of this event. Emerging research has only given considerable attention to the socio-economic factors associated with first premarital childbearing in Africa, its implications for the marriageability of single mothers, and child health [1, 4–6, 11, 14, 20]. While it seems at least plausible to assume that the factors that contributed to having a first premarital birth could warrant the second, understanding the timing of second premarital birth in sub-Saharan African countries is equally important for improving the life chances of single mothers and their children. The short interval between premarital births akin to marital births, not only has implications for maternal and child health [23–27] but could also aggravate the risks associated with having a birth premaritally, since having multiple premarital births implies that single mothers may have to cater for larger families with leaner economic resources. A rapid progression into second premarital childbearing could also be an indication of contraceptive failure, misuse, or non-use and could imply that single mothers, especially those with low socio-economic status, are unable to plan their lives outside of childbearing. For convenience, we use the term premarital childbearing, and premarital births interchangeably to refer to live births before marriage. We also use single mothers to refer to women who have never married.

### Current study

In this article, we draw from the recent scholarly interest in premarital childbearing to elucidate the timing of a second premarital birth among single mothers in sub-Saharan African countries. We anticipated lower rates of second premarital childbearing and differing patterns across countries, especially in countries where premarital childbearing is highly stigmatized for a few reasons. First, emerging studies have highlighted the health returns of premarital childbearing for children and their mothers [7, 22]. As a result, we expect that many single mothers, especially those who are socio-economically disadvantaged, may delay having a second premarital birth and may engage in more protective sexual behaviors to avoid or delay another premarital birth after experiencing the adverse effects of the first premarital birth. Moreover, a recent study conducted in South Africa argues that the failure of family planning programs to target young women before their first pregnancy is associated with higher levels of first premarital childbearing [2]. The authors further showed that contraceptive use is lower before the first birth but increases thereafter, thus reinforcing our hypothesis that single mothers may delay a second premarital birth [2].

We also assess how the timing of second premarital childbearing differs by key socio-demographic characteristics. We hypothesize that the timing of a second premarital birth will differ across key socio-demographic characteristics, mainly because the health disadvantages of premarital childbearing are not evident across all socio-economic spectrums. Secondary or higher levels of maternal education reduce the effect of premarital childbearing on child health and well-being [7, 22]. As a result, a rapid progression to second premarital childbearing among single mothers of high socio-economic status may be an indication of choice rather than an unplanned second premarital birth, since premarital childbearing is not at all disadvantageous to this subpopulation [7].

## Methods

### Study design

In this study, we used cross-sectional data from the recent (2013–2018) Demographic and Health Surveys (DHS) of 25 countries in the sub-Saharan African region. For most countries, the surveys were the sixth or seventh round. The DHS is a nationally representative survey that monitors the demography and health of populations in developing countries. The DHS collects rich information about women’s reproductive histories, including the number of children ever born per woman, their age at the birth of individual births (for women who reported having at least one birth), as well as the month and year of each birth. The women were also asked to give substantial information about their current marital union, including whether they had ever married, and if so, the month and year of their first marriage. The survey’s rich information on women, including their marital status and fertility behaviors, made it a valuable resource for this study.

### Participants and study size

The full data sample for this study comprised of 63,431 women from the selected countries who have had at least one premarital birth at the time of the survey. We considered only women who had their first premarital birth at 15 years or older because the pattern of childbearing at younger ages are likely to differ and could have been biased by the inability of the women to report the date of their first birth accurately. As a result, we excluded about 9% (5853) of the women who were less than 15 years at the time of their first birth. A similar exclusion criterion has been used in a prior study, although they excluded only women with first births before 13 years [5]. We also excluded 221 single mothers (less than 1%) with missing information on the key socio-demographics. Finally, we excluded 138 women who had their first birth less than one month before the survey.

The final analytic sample for our study was 57,219 women who have had at least one premarital birth more than one month before the survey and at 15 years or older and had complete information on the key socio-demographic characteristics considered in this study. The total sample size for each country ranged from 624 women in Ethiopia (East Africa) and the Republic of Chad (625) to 6107 women in Kenya (East Africa). A full list of the countries and their respective sample sizes are presented in Table 1.

**Table 1:** Descriptive Profile of Women with at least one premarital birth in 25 sub-Saharan African Countries *(Source: Demographic and Health Survey data sets)*

Country (Survey Year)	Percentage Distributions (%)						Total sample
	Had first birth at 25+ years	Residing in an urban place of residence	Can read all words	Attained secondary or higher education	Has a second premarital birth	Married before a second premarital birth	
Central Africa							
Angola 2015–16	6.29	70.75	43.46	43.74	40.29	40.71	4734
Chad 2014–15	3.47	28.94	21.72	20.28	29.71	55.08	625
East Africa							
Burundi 2016–17	10.68	19.84	64.36	27.23	19.56	49.33	1221
Congo DRC 2013–14	6.75	44.14	61.55	54.51	27.73	42.31	1822
Ethiopia 2016	10.39	20.19	19.89	9.95	27.58	61.21	624
Kenya 2014	5.84	40.30	82.22	41.16	20.69	58.90	6107
Rwanda 2014–15	11.76	24.92	69.59	21.70	23.54	36.48	1454
Tanzania 2015–16	5.70	40.97	75.59	21.10	24.00	56.39	2188
Uganda 2016	4.28	30.41	57.83	37.38	29.77	53.45	3065
Southern Africa							
Lesotho 2014	8.36	41.83	89.05	60.18	25.23	45.08	1068
Malawi 2015–16	2.75	22.77	66.71	31.52	21.98	61.36	3321
Namibia 2013	11.21	55.77	89.60	75.39	51.88	20.42	4532
South Africa 2016	11.79	65.79	87.20	88.95	47.11	24.05	4667
Zambia 2013–14	3.49	47.42	67.28	54.09	22.98	53.49	3444
Zimbabwe 2015	6.59	35.69	85.49	69.77	26.83	57.01	1497
West Africa							
Ghana 2014	9.38	55.55	41.47	65.92	28.29	45.13	1342
Guinea 2018	9.47	44.68	17.01	23.59	30.24	45.81	1047
Liberia 2013	3.81	64.02	47.81	45.75	35.01	38.14	2464
Sierra Leone 2013	6.72	50.57	43.79	42.99	34.03	33.19	2534
Togo 2013–14	10.87	50.49	41.28	35.66	18.42	60.27	1025
Benin 2017–18	9.76	46.98	26.45	24.30	30.39	54.94	1962
The Gambia 2013	4.40	71.03	51.66	49.91	25.08	49.84	693
Mali 2018	6.33	39.81	27.92	32.37	24.56	60.32	1125
Nigeria 2018	12.66	52.20	33.75	57.50	32.97	51.14	3560
Senegal 2017	12.99	61.60	45.96	33.73	22.32	50.68	1098

### Measurement of variables

#### Dependent variable

The primary outcome variable for this study is the timing of a second premarital birth among women who have had at least one birth before marriage. This variable was measured in years and assessed the difference between the first and second premarital births and may only be censored by not having a second premarital child at the time of the survey. Our analysis also recognized the likelihood of marriage before the second premarital birth and treated this as a competing risk. In such situations, marriage could reduce the likelihood of second premarital birth. Following the practice in this research area, we considered women whose first and second births occurred at least one month before entry into marriage or cohabitation as having a premarital birth (Clark, Koski, Smith-Greenaway, 2017). Similarly, we considered both formal and informal unions to be “marriages” [28].

To assess the timing of the second premarital birth in the presence of a competing risk, we first created a status variable as an indication of whether a woman has had a second premarital birth or not and whether it was before marriage. Combining information on the year of the first and second births, as well as the year of marriage, women who did not have a second premarital birth nor were married at the time of data collection were coded “0”. Women who were single but did have a second premarital birth at the time of the survey or were married but had the second premarital birth before marriage were coded “1”. Finally, women who were married but did not have a second premarital birth at the time of the survey or were married and had the second birth after marriage were coded “2”.

With the classification above, and by adapting the event-history modeling approach, women in the first status category were censored at the time of the survey. This implied that we calculated the interval between the date of the first premarital birth and the date of the survey. For women in the category “1” of the status variable, we estimated the interval between the first and second premarital birth, while we estimated the difference between the date of birth of the first premarital child and the dates of marriage for women in category “2”.

#### Covariates

Our analysis included key socio-demographic characteristics such as women’s age at the birth of the first premarital child, place of residence, educational attainment, and literacy. Women’s age at the birth of the first premarital child was estimated by subtracting the woman’s date of birth from the date of birth of the first premarital child. The difference between both variables (measured in months) was subsequently divided by 12 to obtain a measure of the woman’s age in years. The age was then categorized into “0” for women who had their first premarital birth at a young age (15–24 years) and “1” for women who had their first premarital birth at 25 years or older. Educational attainment was assessed from the question that asked for the highest level of education that a woman had attained at the time of the survey. Responses to this question were dichotomized as “0” for women with less than secondary education (primary or no formal education) and “1” for women with secondary or higher education. This classification is premised on the notion that opportunities for education, at least at the secondary level, could serve as a direct incentive to delay a second premarital birth [29]. Information on literacy was obtained from the question that asked women to read certain words. Women who were able to read all the words were categorized as “1 – can read all,” while those who could only read a part or were unable to read at all were classified as cannot read and coded “0”. Finally, we included a dummy indicator of the place of residence, which we coded as “0” for rural residence and “1” for urban.

### Statistical analysis

Frequency and percentage distributions are used to describe key socio-demographic characteristics of women in the sample across the 25 sub-Saharan African countries.

Our analyses also adopted a competing risk model framework to estimate the probability of having a second premarital birth in the presence of a competing event (marriage after the first premarital birth) and whether it is associated with key socio-demographic characteristics. Our competing risk framework is a special form of survival analysis in which another event (marriage) inhibits the occurrence of the event of interest (second premarital birth).

Although several methods of estimating cumulative incidence in the presence of competing risk exists [30, 31], our analysis adopted Fine and Gray’s subdistribution hazard model [32] in understanding women’s probability of having a second premarital birth conditional on covariates and considers that women who marry after the first premarital birth will never have a second premarital birth. The choice of this model is based on its acceptability and prior empirical applications as demonstrated by Austin and Fine [33] as well as Wolbers et al. [34]. An elegant explanation of the Fine and Gray model has been published in another study [35]. While its application has mostly been in medical and epidemiological research, its application in studying population processes such as fertility, mortality, and family formation has also been increasing [36–40].

Using Fine and Gray’s subdistribution hazard model, we estimated the probabilities of having a second premarital birth among women in each of the countries sampled. Cumulative incidence graphs were used to visualize the incidence of a second premarital birth across countries. In the second part of our analysis, we fitted a multivariate competing risk regression model to identify socio-demographic differentials in the incidence of a second premarital birth.

The interpretation of the results was made using subdistribution hazard ratios (SHR), which is the relative change in the subdistribution hazard function. Unlike other models of competing risk analysis, the regression coefficients obtained from Fine and Gray’s model are directly linked to the cumulative incidence function (CIF), and the occurrence of competing events influences the coefficients [41]. The subdistribution hazard function enabled us to estimate the effect of covariates on the cumulative incidence function for the event of interest. As recommended by Austin and Fine [33], the magnitude of the subdistribution hazard ratio denotes the direction but not directly the magnitude of the effect of the covariate on the CIF. As a result, we interpreted an SHR > 1 implied a higher incidence or risk, SHR < 1 implied a lower incidence or risk, and SHR = 1 implied no difference in incidence or risk. All analyses were performed with the use of Stata statistical software version 14.

## Results

### Descriptive profile of the participants

Table 1 presents the descriptive profile of women in the sample by key socio-demographics. More than one-quarter of women in 16 of the countries studied had at least two premarital births, while more than half of the women who had at least one premarital birth in 13 countries were married before the birth of their second child. Across key socio-demographics, only about one in 10 women had their first premarital birth at or after 25 years. In Angola, Namibia, South Africa, Ghana, Liberia, Sierra Leone, Togo, the Gambia, Nigeria, and Senegal, more than half of the women in the sample resided in an urban place of residence. In all West African countries (except the Gambia) and Central African countries in this study, less than half of the women, could read all the words, while more than half of the women in the Southern African countries could read all the words. In most of the Southern African countries in the study sample, more than half of the women had attained secondary or higher education, while only half of the women in the Republic of Congo, Ghana, and Nigeria had attained secondary or higher levels of education in the East and West African regions.

### Timing of second premarital births among single-mothers in 25 sub-Sahara African countries

**Fig 1:**
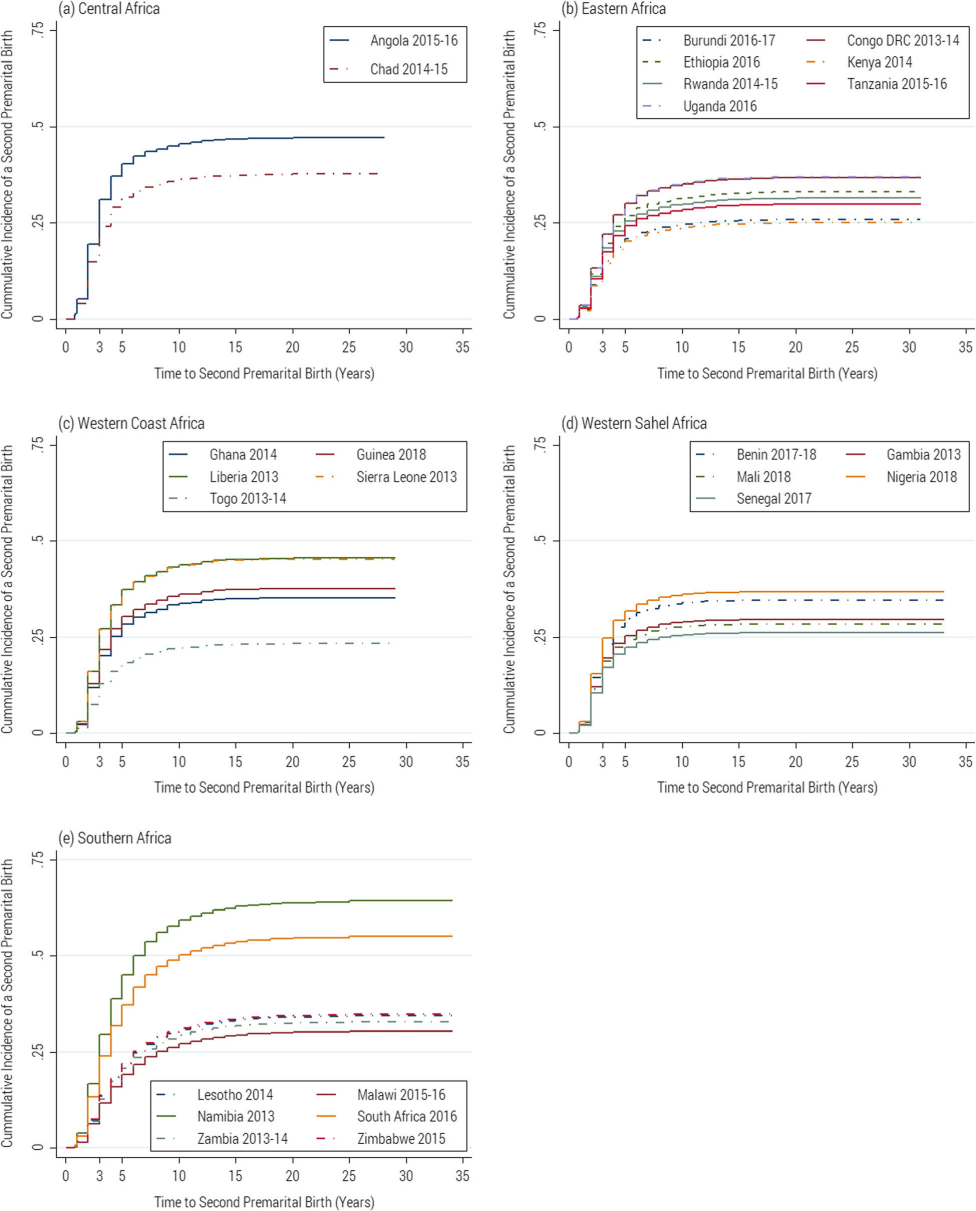
Cumulative Incidence of Second Premarital Births by Region and Countries among women in 25 countries in sub-Saharan Africa

The cumulative incidence of a second premarital birth by countries and regions are shown in Fig. 1. Across the countries, the results show that more than one-third of single mothers in most of the countries have had a second premarital birth in their reproductive life. The results also show that more than 15% of the single mothers in Angola, Benin, the Republic of Chad, Liberia, Namibia, Nigeria, Sierra Leone, and Uganda, have had another premarital birth by three years. In all countries, more than two-thirds of women who have had a second premarital birth had them within the first five years.

### Correlates of a second premarital birth

Table 2 shows the results from the competing risk regression models for each country and highlights statistically significant differences in the timing of a second premarital birth by key socio-demographic variables. The most notable difference in the timing of a second premarital birth was observed across the levels of education. In most of the countries studied, the incidence of a second premarital birth differed by education attainment (*p < 0.05*). Women who had secondary or higher education had a lower risk of a second premarital birth, compared to women with less than secondary education.

**Table 2:** Sub-hazard ratios and 95% confidence intervals comparing the incidence of a second premarital birth by key socio-demographic characteristics among 57,219 women in Sub-Saharan African countries

Country (Survey Year)	Sub-Hazard Ratios [95% Confidence Intervals]				
	Had first birth at 25+ years	Residing in an urban place of residence	Can read all words	Attained secondary or higher education	Total sample
	15–24^‡^ vs 25 years +	Rural^‡^ vs Urban	Can’t read all^‡^ vs Can read all words	<Secondary^‡^ vs Secondary+	
Central Africa					
Angola 2015–16	0.94 [0.77,1.15]	0.91 [0.81,1.03]	0.84* [0.72,0.99]	0.60*** [0.51,0.72]	4734
Chad 2014–15	0.70 [0.29,1.69]	1.01 [0.67,1.53]	1.36 [0.44,4.22]	0.29* [0.09,0.97]	625
East Africa					
Burundi 2016–17	0.96 [0.63,1.45]	0.85 [0.57,1.25]	0.75 [0.55,1.02]	0.59* [0.39,0.89]	1221
Congo DRC 2013–14	0.79 [0.49,1.27]	1.04 [0.81,1.35]	1.40 [0.85,2.30]	0.54* [0.33,0.88]	1822
Ethiopia 2016	1.07 [0.51,2.24]	0.70 [0.40,1.22]	0.61 [0.31,1.19]	1.42 [0.61,3.30]	624
Kenya 2014	1.10 [0.81,1.51]	0.85* [0.73,0.99]	0.84* [0.71,0.98]	0.70*** [0.59,0.82]	6107
Rwanda 2014–15	0.81 [0.56,1.18]	0.62*** [0.47,0.82]	0.94 [0.75,1.19]	0.55** [0.39,0.80]	1454
Tanzania 2015–16	1.51* [1.02,2.22]	1.00 [0.82,1.23]	0.80* [0.65,0.99]	0.44*** [0.31,0.63]	2188
Uganda 2016	1.26 [0.88,1.79]	1.07 [0.90,1.28]	0.79** [0.67,0.94]	0.59*** [0.47,0.72]	3065
Southern Africa					
Lesotho 2014	0.99 [0.59,1.68]	0.79 [0.58,1.07]	0.80 [0.55,1.18]	0.75 [0.55,1.02]	1068
Malawi 2015–16	1.09 [0.65,1.83]	1.16 [0.89,1.50]	0.68*** [0.56,0.83]	0.695** [0.54,0.90]	3321
Namibia 2013	0.62*** [0.53,0.74]	0.69*** [0.64,0.76]	1.06 [0.91,1.23]	0.655*** [0.59,0.73]	4532
South Africa 2016	0.72*** [0.59,0.87]	0.76*** [0.69,0.84]	0.82** [0.70,0.95]	0.67*** [0.58,0.78]	4667
Zambia 2013–14	1.04 [0.63,1.72]	0.78** [0.65,0.93]	0.965 [0.76,1.23]	0.79 [0.62,1.02]	3444
Zimbabwe 2015	0.64 [0.37,1.11]	1.04 [0.80,1.34]	0.87 [0.65,1.17]	0.74* [0.57,0.96]	1497
West Africa					
Ghana 2014	0.67 [0.42,1.08]	0.79* [0.62,1.00]	1.00 [0.74,1.35]	0.76* [0.57,0.99]	1342
Guinea 2018	0.80 [0.53,1.22]	1.09 [0.86,1.39]	0.80 [0.42,1.51]	0.665 [0.39,1.13]	1047
Liberia 2013	0.54* [0.31,0.93]	0.88 [0.75,1.05]	1.12 [0.63,1.97]	0.75 [0.42,1.35]	2464
Sierra Leone 2013	1.31 [0.96,1.78]	1.03 [0.87,1.22]	0.66 [0.22,1.96]	1.04 [0.35,3.12]	2534
Togo 2013–14	1.3 [0.82,2.09]	1.13 [0.82,1.56]	0.58 [0.29,1.14]	1.12 [0.55,2.30]	1025
Benin 2017–18	1.00 [0.75,1.33]	0.76** [0.64,0.91]	0.79 [0.58,1.08]	0.78 [0.56,1.08]	1962
The Gambia 2013	1.01 [0.41,2.49]	1.37 [0.91,2.08]	0.53 [0.079,3.50]	1.33 [0.20,8.79]	693
Mali 2018	0.58 [0.33,1.04]	1.20 [0.92,1.56]	0.78 [0.52,1.18]	0.80 [0.54,1.18]	1125
Nigeria 2018	0.72** [0.57,0.91]	0.77*** [0.67,0.89]	0.88 [0.73,1.07]	0.67*** [0.56,0.80]	3560
Senegal 2017	0.86 [0.53,1.41]	0.72* [0.54,0.97]	0.87 [0.54,1.38]	0.90 [0.53,1.51]	1098

***p < .01; **p < .05; *p < .1.^‡^ − *Denotes reference category*

In Tanzania [SHR: 1.51, 95% CI: 1.02,2.22], the risk of a second premarital birth was higher for women who had their first premarital birth after 25 years, compared to those who had their first premarital birth between 15 and 24 year. In Namibia [SHR: 0.62, 95% CI: 0.53,0.74], South Africa [SHR: 0.72, 95% CI: 0.59,0.87], Liberia [SHR: 0.54, 95% CI: 0.31,0.93], and Nigeria [SHR: 0.72, 95% CI: 0.57,0.91], the incidence of a second premarital birth was lower for women who had a first premarital birth at age 25 years or more, compared to 15–24 years. Residence in an urban place of residence was also associated with a low incidence of a second premarital birth in Kenya [SHR: 0.85, 95% CI: 0.73,0.99], Rwanda [SHR: 0.62, 95% CI: 0.47,0.82], Namibia [SHR: 0.69, 95% CI: 0.64,0.76], South Africa [SHR: 0.76, 95% CI: 0.69,0.84], Zambia [SHR: 0.78, 95% CI: 0.65,0.93], Ghana [SHR: 0.79, 95% CI: 0.62,1.00], Benin [SHR: 0.76, 95% CI: 0.64,0.91], Nigeria [SHR: 0.77, 95% CI: 0.67,0.89], and Senegal [SHR: 0.72, 95% CI: 0.54,0.97]. The ability to read was not associated with the incidence of second premarital birth in all West African countries, but a significant association was observed in Angola, Kenya, Tanzania, Uganda, Malawi, and South Africa.

## Discussion

In this study, we examined the timing of a second premarital birth among single mothers in 25 sub-Saharan African countries. We were able to delineate country-level and regional variations in the progression to a second premarital birth among single mothers. The results from this study extend and expand on previous research in two ways. First, our study is one of the first to show that families formed outside of marriage are increasing, not only in number but also in size. Our analysis also highlights how long single mothers wait before they have a second premarital birth.

Contrary to the expectation that contraceptive use increases after the first premarital birth [2] and single mothers may be able to delay a second premarital birth as a result, our analysis suggests otherwise in a few countries. On average, the time to a second premarital birth was shorter in most of Western Sahel, Eastern, and Southern Africa, than in the Central African countries. This pattern is similar to those observed for the first premarital birth in the region [1]. Some studies have attributed the variations across countries to increased normative acceptance of single motherhood, which may have reduced the associated costs, sanctions, and stigma around premarital childbearing in some of the countries [11, 42, 43].

Second, our analysis provides evidence that the incidence of a second premarital birth differs significantly by women’s educational attainment in most countries. In a few countries, we also observed that the timing of a second premarital birth is lower among younger women, those in rural areas, and women who are not literate. This finding contrasts with our hypothesis, considering that lower levels of education and high levels of unemployment place many single mothers in precarious positions, struggling to pay for food, and other necessities critical for the health and well-being of themselves and their first child [18]. Although understanding why single mothers have a second premarital birth despite its associated negative consequences is beyond the scope of this study, one plausible explanation is that socio-economically disadvantaged single mothers may have low negotiating power and lack access to modern contraceptive methods that could help minimize the risk of a second birth [42, 44, 45].

As families formed outside of marriage continue to increase in size in sub-Saharan African countries, especially in Angola, Sierra Leone, Liberia, South Africa, Namibia, and other countries where a significant proportion of single mothers have had another premarital birth, there is a need to review the situation. Children living in such contexts require high levels of supervision and attention since the mothers are more likely to be young, unlikely to have secondary or higher education, have higher poverty rates, and fewer economic resources than married mothers [16–18]. Evidence from sub-Saharan African countries has demonstrated the poor health returns associated with having a single mother with less than secondary education [7, 22]. Poverty being an associated factor of single motherhood is also related to poor nutrition and low rates of immunization [46, 47].

Though further research is needed to understand why socio-economically disadvantaged single mothers have another premarital birth and rapidly in a few countries, the findings of this study are also relevant for future research on premarital childbearing in sub-Saharan African countries. It is imperative to explore whether different men father the two premarital births. This may particularly be of interest to policymakers in the family planning and HIV prevention programs, as it may reflect the level of women’s susceptibility to sexually transmitted infections in the absence of effective means of contraception, especially condoms. We also anticipate that our study will be followed by future work that seeks to understand how socio-economic disadvantage and social structures, including stigma, serve as facilitators of a second premarital birth.

Our study, being one of the first to examine the timing of a second premarital birth, has a few weak points. First is the cross-sectional nature of the data, and as a result, we cannot completely rule out the possibility of a reverse causality whereby low education may result in having another premarital birth, but having a second premarital birth may also contribute to delays in the educational advancement of the single mother especially where premarital childbearing is socially unacceptable and stigmatized. However, there is a very minimal possibility of reverse causality with other study variables like age at first birth, place of residence, and perhaps literacy. Secondly, ensuring that women accurately report the date of their marriage is challenging in any setting and may be especially so in African contexts, where the formalization of unions often involves customs that occur over several months [28, 48, 49]. Even more challenging is the likelihood of under-reporting a premarital birth, especially in religious communities, where having a child before marriage is strongly stigmatized [4, 50, 51]. In these communities, women may be more likely to misreport the timing of their first birth and marriage to avoid disclosing a premarital birth [5]. As a result, our sample could have underestimated the rate of premarital births. Despite these limitations, our study offers some insightful perspectives on the demography of premarital childbearing in sub-Saharan African countries.

## Conclusions

Overall, our study found a mixed pattern of progression to having a second premarital birth in sub-Saharan Africa. We noted the rapid succession of first premarital births in Angola, Benin, the Republic of Chad, Liberia, Namibia, Nigeria, Sierra Leone, and Uganda, where more than 15% of single mothers have had another child before marriage by the third year. The timing of a second premarital birth differed significantly by educational attainment in most countries while residing in an urban area and being literate was associated with a lower incidence of second premarital birth in a few countries.

## Data Availability

The datasets supporting the conclusions of this article are available on the demographic and health survey website (https://dhsprogram.com/data/available-datasets.cfm).

## Abbreviations

CIF: Cumulative incidence function
DHS: Demographic and Health Surveys
S.E: Standard error
SHR: Sub-hazard ratio
SSA: sub-Saharan Africa(n)
